# Family history of prostate cancer and prostate tumor aggressiveness in black and non-black men;results from an equal access biopsy study

**DOI:** 10.1007/s10552-020-01389-8

**Published:** 2021-02-02

**Authors:** Kimberly R. Jenkins, Taofik Oyekunle, Lauren E. Howard, Emily K. Wiggins, Stephen J. Freedland, Emma H. Allott

**Affiliations:** 1 Division of Urology, Veterans Affairs Medical Center, Durham, NC USA; 2 Duke Cancer Institute, Duke University School of Medicine, Durham, NC USA; 3 Center for Integrated Research on Cancer and Lifestyle, Cedars-Sinai Medical Center, Samuel Oschin Comprehensive Cancer Institute, Los Angeles, CA USA; 4 Patrick G. Johnston Centre for Cancer Research, Queen’s University Belfast, 97 Lisburn Road, Room 2.15, Belfast, BT9 7AE UK; 5 Department of Histopathology and Morbid Anatomy, Trinity Translational Medicine Institute, Trinity College Dublin, Dublin, Ireland

**Keywords:** African American, Family history of prostate cancer, Prostate biopsy, Prostate cancer, Tumor grade

## Abstract

**Purpose:**

To test for racial differences in associations between family history (FH) of prostate cancer (PC) and prostate cancer aggressiveness in a racially diverse equal access population undergoing prostate biopsy.

**Subjects/patients and methods:**

We prospectively enrolled men undergoing prostate biopsy at the Durham Veterans Administration from 2007 to 2018 and assigned case or control status based on biopsy results. Race and FH of PC were self-reported on questionnaires. Logistic regression was used to test the association between FH and PC diagnosis overall and by tumor aggressiveness [high- (Grade Group 3–5) or low-grade (Grade Group 1–2) vs. no cancer], overall, and stratified by race. Models were adjusted for age and year of consent, race, PSA level, digital rectal exam findings, prostate volume, and previous (negative) biopsy receipt.

**Results:**

Of 1,225 men, 323 had a FH of PC and 652 men were diagnosed with PC on biopsy. On multivariable analysis, FH was associated with increased odds of high-grade PC in black (OR 1.85, *p* = 0.041) and all men (OR 1.56, *p* = 0.057) and was unrelated to overall or low-grade PC diagnosis, overall, or stratified by race (all *p* ≥ 0.325). In sensitivity analyses among men without a previous biopsy, results were slightly more pronounced.

**Conclusion:**

In this setting of equal access to care, positive FH of PC was associated with increased tumor aggressiveness in black men, but not non-black men undergoing prostate biopsy. Further research is required to tease apart the contribution of genetics from increased PC awareness potentially influencing screening and biopsy rates in men with FH.

**Supplementary Information:**

The online version of this article (10.1007/s10552-020-01389-8) contains supplementary material, which is available to authorized users.

## Introduction

Approximately 20% of all new cancer cases in the USA in 2019 are expected to be due to prostate cancer, affecting an estimated 174,650 men. Additionally, prostate cancer is projected to account for around 10% of all US cancer deaths [1]. For black men during the same time period, it is expected that prostate cancer will account for approximately 30% of all new cancer cases and 15% of all cancer deaths, one of the largest racial disparities of any cancer type [2]. Even when adjusting for clinical and socioeconomic risk factors, black men have a disproportionate burden of aggressive prostate cancer [3], suggesting that biological factors may also contribute.

Family history of prostate cancer is an established risk factor for prostate cancer [4] and the disease has previously been found to have a high estimate of heritability of 57% in a white European population [5]. Many previous studies have explored prostate cancer risk in men with a family history of the disease in predominantly white study populations [4–7]. Whether these findings apply or not to black men is not clear. Only four studies, to our knowledge, have examined risk of overall prostate cancer in both black and white men with a family history of the disease and they found no substantial differences in associations by race [8–12]. However, these previous studies did not consider tumor aggressiveness. Of studies examining tumor aggressiveness, two found that family history of prostate cancer was associated with increased risk of fatal prostate cancer [6, 13], though these studies were limited to white men. Finally, a study including < 2% black men found that family history of prostate cancer was associated with an increased overall prostate cancer risk, as well as low-grade and high-grade prostate cancer risk [4]. There were not, however, enough black men in the study to test for race interactions by tumor aggressiveness.

Given the excess burden of aggressive and fatal prostate cancer in black men, we hypothesized that a positive family history of prostate cancer would be more strongly associated with prostate tumor aggressiveness in black men than non-black men. To test this, we prospectively enrolled men undergoing a prostate biopsy at the Durham Veterans Affairs Medical Center (DVAMC) in Durham, North Carolina. The current analysis uses data from a case–control study nested within this prospective cohort of men undergoing prostate biopsies. This location serves a racially diverse patient population to allow for exploration of racial differences in associations between family history of prostate cancer and prostate cancer aggressiveness in the context of equal access to care.

## Materials and methods

### Study design

Men undergoing prostate biopsy for an elevated PSA and/or abnormal digital rectal examination (DRE) at the DVAMC from January 2007 to October 2018 were prospectively enrolled. Methods for identification and accrual of participants have been described previously [14]. Men were at least 18 years of age, had a PSA test within 12 months prior to enrollment, and had no history of prostate cancer. A total of 1,313 men meeting these eligibility criteria undergoing biopsy were enrolled between January 2007 and October 2018. We excluded 70 men due to previous positive biopsies. Of the remaining 1,243 men, we excluded 18 for missing data on body mass index (BMI), biopsy result, DRE, transurethral ultrasound (TRUS) prostate volume or Grade Group, resulting in a study cohort of 1,225 patients. The current analysis uses data from a case–control study nested within this prospective cohort of men undergoing prostate biopsies. Of the 1,225 men who underwent a biopsy and were included in the analysis, 652 (53%) were biopsy positive (cases) and 573 (47%) were biopsy negative (controls). The study was approved by the Institutional Review Board at the DVAMC and all patients provided written informed consent.

### Data collection

Patients completed a questionnaire, including demographic, medical, and lifestyle characteristics. In the family history section of the questionnaire, patients were prompted to “indicate the number of blood relatives you have who have been diagnosed with prostate cancer.” A chart was included below this question with the following categories of blood relatives: father, grandfathers, great grandfathers, brother(s), half-brother(s), son(s), uncle(s), great uncle(s), first cousin(s), and nephew(s). The response categories for each blood relative were yes, no, don’t know, and N/A. In instances where this section was left blank, or only “don’t know” and/or “N/A” were indicated, the family history of prostate cancer for that patient was categorized as unknown. Patients who responded yes to any blood relative were classified as having a family history of prostate cancer.

Affected blood relatives were categorized as first-degree [father, son(s), brother(s)] or second-degree only [half-brother(s), uncle(s), great uncle(s), first-degree cousin(s), nephew(s)]. For men verbally reporting a family history of prostate cancer during the consenting process at the clinic, but who did not indicate the affected relative on the questionnaire (n = 90), a chart review was conducted, and family history degree data were abstracted if available (*n* = 80). We categorized the remaining 10 patients as having a second-degree family history of prostate cancer, since this was more likely given the patients’ lack of knowledge regarding which specific relative had a diagnosis of prostate cancer in the past.

All questionnaires were self-administered and typically filled out shortly after the biopsy procedure, but prior to patients being aware of their biopsy results, and returned by mail. Race was self-reported on questionnaires and categorized as black vs. non-black (> 98% white). Anthropometric measurements (measured weight and height, used to calculate BMI), DRE findings, prostate volume, and PSA level were abstracted from urology clinic notes from either the visit at which biopsy was performed, or the most recent visit prior to biopsy.

### Outcome ascertainment

Biopsy tissue was assessed by a pathologist per standard of care. Among those with a positive biopsy, prostate cancer grade was abstracted from the resulting pathology report. Grade was assigned using Epstein’s five-Grade Group system where low-grade disease was defined as Grade Group 1–2 (Gleason score ≤ 3 + 4) and high-grade prostate cancer as Grade Group 3–5 (Gleason score ≥ 4 + 3) [15].

### Statistical analysis

Demographic and clinical characteristics of men undergoing prostate biopsy with positive, negative, or unknown family history of prostate cancer were compared using Kruskal–Wallis and chi-square tests for continuous and categorical variables, respectively. Logistic regression was used to test the association between family history of prostate cancer (positive, negative, or unknown) and odds of overall prostate cancer diagnosis on biopsy. Similarly, multinomial logistic regression was used to test the association between family history of prostate cancer and tumor aggressiveness [high-grade (Grade Group 3–5) or low-grade (Grade Group 1–2) vs. no cancer]. Both age-adjusted and multivariable-adjusted models were considered. Multivariable-adjusted models included age, year of consent, race (black vs. non-black), PSA (log transformed), DRE findings (normal vs. suspicious), prostate volume (log transformed), and previous (negative) biopsy (yes vs. no). Given that our outcomes of overall, low-grade, and high-grade prostate cancer do not fit the rare disease assumption, odds ratios (ORs) should not be interpreted as relative risk ratios.

In sub-analysis, positive family history of prostate cancer was further categorized as first degree or second degree, treating men with no family history of prostate cancer as the referent group and omitting men with unknown family history. Men reporting both first- and second-degree family history were classified as having first-degree family history of prostate cancer.

All analyses were conducted among all men and stratified by race. Further, we tested for interactions between family history of prostate cancer and race by including both main effect terms and an interaction term, which represented the cross-product of the two main effect terms in the same model. The coefficient of the cross-product term was tested by the Wald test.

In sensitivity analysis, we restricted models to men without a previous negative prostate biopsy.

Analyses were performed using SAS 9.4 (SAS Institute, Inc. Cary, NC). Statistical significance was two-sided with a threshold of *p* < 0.05.

## Results

### Patient characteristics

Of 1,225 men included in the analysis, 323 (26%) reported a family history of prostate cancer, 627 (52%) reported no family history of prostate cancer, and 275 (22%) did not know if they had a family history of prostate cancer (Table 1). Median (IQR) age at biopsy did not differ significantly by family history status (*p* = 0.083). Men with a positive family history tended to be consented in more recent years, relative to those with unknown or no family history (*p* < 0.001). Median PSA was similar across categories of family history, as was median prostate volume and BMI. Men with a positive family history had slightly higher rates of previous (negative) biopsy, relative to those without a family history of prostate cancer (21% vs. 17%), though biopsy rates were not significantly different across all three categories of family history (*p* = 0.410). Frequency of previous negative prostate biopsy did not differ by race (19% of black men vs. 18% of non-black men; chi-square *p* = 0.456), neither did the frequency of family history vary by race (27% black men vs. 25% non-black men, *p* = 0.316). Among men with family history, the degree of family history did not vary by race (*p* = 0.270), with 74% first-degree among black men and 80% first-degree among non-black men.

**Table 1:** Characteristics of men undergoing prostate biopsy by family history status

	Total (*N* = 1,225)	Family history of prostate cancer			
		Yes (*N* = 323)	No (*N* = 627)	Unknown (*N* = 275)	*p*-value*
Age at consent					0.083^a^
Median	63	64	64	62	
(IQR)	(59, 68)	(60, 68)	(59, 68)	(59, 67)	
Race					0.316^b^
Non-black	508 (42)	128 (40)	273 (44)	107 (39)	
Black	717 (58)	195 (60)	354 (56)	168 (61)	
Year of consent					< 0.001^a^
Median	2011	2014	2011	2010	
(IQR)	(2009, 2016)	(2009, 2016)	(2008, 2016)	(2009, 2014)	
PSA at biopsy (ng/mL)					0.131^a^
Median	6.1	5.9	6.0	6.4	
(IQR)	(4.7, 8.6)	(4.5, 8.4)	(4.6, 8.4)	(4.9, 9.4)	
Digital rectal exam					0.082^b^
Not suspicious for cancer	925 (75)	233 (72)	472 (75)	220 (80)	
Suspicious for cancer	300 (25)	90 (28)	155 (25)	55 (20)	
TRUS prostate volume (cc)					0.474^a^
Median	42.0	42.0	42.0	40.6	
(IQR)	(29.9, 61.0)	(30.8, 61.5)	(29.0, 61.0)	(29.0, 61.8)	
BMI (kg/m^2^)					0.199^a^
Median	29.1	29.7	29.1	28.6	
(IQR)	(26.1, 32.9)	(26.1, 33.7)	(26.2, 32.9)	(25.4, 32.3)	
Previous negative biopsy					0.410^b^
No	998 (81)	256 (79)	519 (83)	223 (81)	
Yes	227 (19)	67 (21)	108 (17)	52 (19)	
Cancer on current biopsy					0.633^b^
No Cancer	573 (47)	144 (45)	300 (48)	129 (47)	
Cancer	652 (53)	179 (55)	327 (52)	146 (53)	
Cancer Grade					0.094^b^
No Cancer	573 (47)	144 (45)	300 (48)	129 (47)	
Low-grade	468 (38)	116 (36)	239 (38)	113 (41)	
High-grade	184 (15)	63 (19)	88 (14)	33 (12)	

*PC* prostate cancer, *PSA* prostate-specific antigen, *BMI* body mass index, *TRUS* transrectal ultrasound

*p-values compare characteristics according to family history status (yes, no, unknown)

^a^Kruskal–Wallis

^b^Chi-Square

### Family history and prostate cancer

Overall, 652 (53%) men were diagnosed with prostate cancer at biopsy, of which 184 (28%) had high-grade prostate cancer and 468 (72%) had low-grade prostate cancer. Among all men, family history of prostate cancer was not associated with either overall or low-grade prostate cancer on either age-adjusted or multivariable-adjusted analysis (Tables 2, 3). However, family history of prostate cancer was associated with increased odds of high-grade disease on age-adjusted analysis [Odds Ratio (OR) 1.52, 95% CI 1.04–2.23, *p* = 0.03] with a similar magnitude of association observed following multivariable adjustment (OR 1.56, 95% CI 0.99–2.46, *p* = 0.057). After stratification by race, family history of prostate cancer remained unassociated with overall and low-grade prostate cancer among both black men and non-black men, in both age-adjusted and multivariable analysis. However, family history of prostate cancer was more strongly associated with increased odds of being diagnosed with a high-grade prostate cancer in black men (OR 1.85, 95% CI 1.03–3.34, *p* = 0.04) than in non-black men (OR 1.32; 95% CI 0.63–2.81; *p* = 0.455), though no statistically significant interaction was found between family history of prostate cancer and race in predicting high-grade prostate cancer (*p* ≥ 0.66). Unknown family history of prostate cancer was not associated with overall prostate cancer, low- or high-grade prostate cancer in the entire cohort, or in race-stratified analyses (*p* ≥ 0.50).

**Table 2:** Odds ratios and 95% confidence intervals for associations of family history of prostate cancer with overall prostate cancer diagnosis at biopsy, relative to those with a biopsy negative for prostate cancer, among all men and stratified by race

	Prostate biopsy result			
	Negative	Positive		
	*N*_e_/*N*	*N*_e_/*N*	OR (95% CI)	*p*-value
All men (*n* = 1,225)				
Family History of PC				
No	300/627	327/627	Ref	
Yes	144/323	179/323		
Age-adjusted			1.14 (0.87–1.50)	0.337
Multivariable*			1.17 (0.86–1.60)	0.325
Unknown	129/275	146/275		
Age-adjusted			1.05 (0.79–1.39)	0.749
Multivariable*			1.08 (0.78–1.49)	0.651
Black men (*n* = 717)				
Family History of PC				
No	151/354	203/354	Ref	
Yes	76/195	119/195		
Age-adjusted			1.16 (0.81–1.66)	0.411
Multivariable*			1.21 (0.80–1.83)	0.364
Unknown	68/168	100/168		
Age-adjusted			1.10 (0.76–1.60)	0.607
Multivariable*			1.23 (0.80–1.89)	0.356
Non-Black men (*n* = 508)				
Family History of PC				
No	149/273	124/273	Ref	
Yes	68/128	60/128		
Age-adjusted			1.08 (0.71–1.65)	0.717
Multivariable*			1.18 (0.73–1.91)	0.500
Unknown	61/107	46/107		
Age-adjusted			0.92 (0.58–1.45)	0.713
Multivariable*			0.90 (0.55–1.50)	0.693

*p*-interaction between family history and race in predicting overall PC was 0.657

*PC* prostate cancer, *OR* odds ratio, *CI* confidence interval, *N*_*e*_ number of men with a negative biopsy, or diagnosed with overall prostate cancer, *N* total number of men undergoing prostate biopsy

*Adjusted for age at consent, race (unless stratified by race), year of consent, PSA (log transformed), DRE, TRUS volume (log transformed), and previous negative biopsy

**Table 3:** Odds ratios and 95% confidence intervals for associations of family history of prostate cancer with low-grade and high-grade prostate cancer diagnosis at biopsy, relative to those with a biopsy negative for prostate cancer, among all men and stratified by race

	Prostate biopsy result						
	Negative	Positive: low-grade prostate cancer			Positive: high-grade prostate cancer		
	*N*_*e*_/*N*	*N*_e_/*N*	OR (95% CI)	*p*-value	*N*_e_/*N*	OR (95% CI)	*p*-value
All men (n = 1,225)							
Family history of PC							
No	300/627	239/627	Ref		88/627	Ref	
Yes	144/323	116/323			63/323		
Age-adjusted			1.01 (0.75–1.36)	0.947		1.52 (1.04–2.23)	0.032
Multivariable*			1.08 (0.78–1.50)	0.644		1.56 (0.99–2.46)	0.057
Unknown	129/275	113/275			33/275		
Age-adjusted			1.09 (0.80–1.48)	0.572		0.91 (0.58–1.43)	0.681
Multivariable*			1.10 (0.78–1.53)	0.595		0.99 (0.59–1.69)	0.983
Black men (*n* = 717)							
Family History of PC							
No	151/354	154/354	Ref		49/354	Ref	
Yes	76/195	75/195			44/195		
Age-adjusted			0.97 (0.66–1.43)	0.876		1.78 (1.09–2.92)	0.022
Multivariable*			1.08 (0.70–1.66)	0.729		1.85 (1.03–3.34)	0.041
Unknown	68/168	80/168			20/168		
Age-adjusted			1.15 (0.77–1.70)	0.502		0.94 (0.52–1.71)	0.840
Multivariable*			1.24 (0.80–1.93)	0.341		1.13 (0.56–2.28)	0.728
Non-Black men (*n* = 508)							
Family History of PC							
No	149/273	85/273	Ref		39/273	Ref	
Yes	68/128	41/128			19/128		
Age-adjusted			1.07 (0.67–1.71)	0.789		1.12 (0.60–2.10)	0.721
Multivariable*			1.13 (0.68–1.88)	0.633		1.32 (0.63–2.81)	0.455
Unknown	61/107	33/107			13/107		
Age-adjusted			0.95 (0.58–1.57)	0.847		0.86 (0.42–1.73)	0.662
Multivariable*			0.93 (0.55–1.58)	0.782		0.79 (0.34–1.86)	0.592

*p*-interaction between family history and race in predicting PC aggressiveness was 0.767

*PC* prostate cancer, *OR* odds ratio, *CI* confidence interval, *N*_*e*_ number of men with a negative biopsy, or diagnosed with low-grade or high-grade prostate cancer, *N* total number of men undergoing prostate biopsy

*Adjusted for age at consent, race (unless stratified by race), year of consent, PSA (log transformed), DRE, TRUS volume (log transformed), and previous negative biopsy

### First- and second-degree family history and prostate cancer

Among all men, relative to those with no family history, neither first- nor second-degree family history of prostate cancer was associated with overall or low-grade prostate cancer diagnosis (*p* ≥ 0.14, Tables 4, 5). However, while second-degree family history was unrelated to high-grade prostate cancer, first-degree family history of prostate cancer was associated with increased odds of high-grade prostate cancer (OR 1.86 95% CI 1.12–3.07, *p* = 0.016). In race-stratified analyses, similar magnitudes of association between first-degree family history of prostate cancer and increased odds of high-grade prostate cancer were observed in black (OR 1.85 95% CI 0.96–3.54, *p* = 0.065) and non-black men (OR 2.11; 95% CI 0.92–4.98; *p* = 0.077). In black men, associations between second-degree family history and high-grade prostate cancer were similar to those for first-degree family history. However, among non-black men, second-degree family history of prostate cancer was associated with reduced odds of overall prostate cancer diagnosis (OR 0.29, 95% CI 0.10–0.84, *p* = 0.02), with similar associations for both low-grade (OR 0.29, 95% CI 0.09–0.95, *p* = 0.04) and high-grade prostate cancer (OR 0.24, 95% CI 0.04–1.51; *p* = 0.129), though results for high-grade prostate cancer were not significant.

**Table 4:** Odds ratios and 95% confidence intervals for associations of degree of family history of prostate cancer with overall prostate cancer diagnosis at biopsy, relative to those with a biopsy negative for prostate cancer, among all men and stratified by race

	Prostate biopsy result			
	Negative	Positive		
	*N*_e_/*N*	*N*_e_/*N*	OR (95% CI)	*p*-value
All men (n = 950)				
Family history degree				
None	300/627	327/627	Ref	
First	105/247	142/247		
Age-adjusted			1.25 (0.93–1.69)	0.138
Multivariable*			1.34 (0.95–1.89)	0.094
Second	39/76	37/76		
Age-adjusted			0.85 (0.53–1.38)	0.512
Multivariable*			0.76 (0.43–1.34)	0.350
Black men (n = 549)				
Family history degree				
None	151/354	203/354	Ref	
First	57/145	88/145		
Age-adjusted			1.15 (0.77–1.70)	0.501
Multivariable*			1.18 (0.75–1.86)	0.480
Second	19/50	31/50		
Age-adjusted			1.19 (0.64–2.19)	0.583
Multivariable*			1.28 (0.62–2.67)	0.502
Non-Black men (n = 401)				
Family history degree				
None	149/273	124/273	Ref	
First	48/102	54/102	1.43 (0.90–2.27)	0.133
Age-adjusted			1.68 (0.99–2.85)	0.054
Multivariable*				
Second	20/26	6/26		
Age-adjusted			0.33 (0.13–0.86)	0.023
Multivariable*			0.29 (0.10–0.84)	0.023

*p*-interaction between degree of family history of PC and race in predicting overall PC was 0.039

*PC* prostate cancer, *OR* odds ratio, *CI* confidence interval, *N*_*e*_ number of men with a negative biopsy, or diagnosed with overall prostate cancer, *N* total number of men undergoing prostate biopsy

*Adjusted for: Age at consent, race (unless stratified by race), year of consent, PSA (log transformed), DRE, TRUS volume (log transformed), and previous negative biopsy

**Table 5:** Odds ratios and 95% confidence intervals for associations of degree of family history of prostate cancer with low-grade and high-grade prostate cancer diagnosis at biopsy, relative to those with a biopsy negative for prostate cancer, among all men and stratified by race

	Prostate biopsy result						
	Negative	Positive: Low-grade prostate cancer			Positive: High-grade prostate cancer		
	*N*_e_/*N*	*N*_e_/*N*	OR (95% CI)	*p*-value	*N*_e_/*N*	OR (95% CI)	*p*-value
All men (*n* = 950)							
Family history degree							
None	300/627	239/627	Ref		88/627	Ref	
First	105/247	93/247			49/247		
Age-adjusted			1.12 (0.80–1.55)	0.514		1.66 (1.09–2.52)	0.018
Multivariable*			1.23 (0.86–1.76)	0.252		1.86 (1.12–3.07)	0.016
Second	39/76	23/76			14/76		
Age-adjusted			0.74 (0.43–1.27)	0.269		1.17 (0.60–2.26)	0.652
Multivariable*			0.71 (0.39–1.29)	0.258		0.98 (0.43–2.22)	0.957
Black men (*n* = 549)							
Family history degree							
None	151/354	154/354	Ref		49/354	Ref	
First	57/145	56/145			32/145		
Age-adjusted			0.96 (0.63–1.48)	0.862		1.75 (1.02–3.01)	0.044
Multivariable*			1.05 (0.65–1.69)	0.840		1.85 (0.96–3.54)	0.065
Second	19/50	19/50			12/50		
Age-adjusted			0.97 (0.50–1.91)	0.935		1.85 (0.83–4.12)	0.130
Multivariable*			1.14 (0.53–2.45)	0.732		1.92 (0.72–5.17)	0.196
Non-Black men (*n* = 401)							
Family history degree							
None	149/273	85/273	Ref		39/273	Ref	
First	48/102	37/102			17/102		
Age-adjusted			1.39 (0.84–2.31)	0.202		1.51 (0.77–2.96)	0.230
Multivariable*			1.59 (0.92–2.76)	0.098		2.11 (0.92–4.98)	0.077
Second	20/26	4/26			2/26		
Age-adjusted			0.33 (0.11–1.01)	0.053		0.33 (0.07–1.50)	0.152
Multivariable*			0.29 (0.09–0.95)	0.040		0.24 (0.04–1.51)	0.129

*Adjusted for: Age at consent, race (unless stratified by race), year of consent, PSA (log transformed), DRE, TRUS volume (log transformed), and previous negative biopsy

*p*-interaction between degree of family history of PC and race in predicting PC aggressiveness was 0.132

*PC* prostate cancer, *OR* odds ratio, *CI* confidence interval, *N*_*e*_ number of men with a negative biopsy, or diagnosed with low-grade or high-grade prostate cancer, *N* total number of men undergoing prostate biopsy

Findings were similar, albeit slightly stronger, among men without a previous negative prostate biopsy (Supplemental Tables 1, 2).

## Discussion

It is now recognized that prostate cancer is among the most heritable cancer types [16]. Evidence for the large heritability component of this disease, however, mainly comes from white populations of European descent and less is understood regarding how family history of prostate cancer in black men affects prostate cancer risk. Moreover, despite established clinical and biological heterogeneity of prostate cancer, relatively few studies have considered tumor aggressiveness when analyzing family history. Using data from a racially diverse cohort of men undergoing prostate biopsy, we found that a family history of prostate cancer was associated with increased odds of high grade, but not overall prostate cancer diagnosis. In analyses stratified by race, we found that the magnitude of this association was stronger and reached statistical significance only in black men. Our study is one of the few to examine associations between family history of prostate cancer and tumor aggressiveness in a racially diverse population and could inform our understanding of the relationship of prostate cancer family history to prostate cancer aggressiveness in black and non-black men.

Despite evidence for a stronger association between positive family history and high-grade prostate cancer in black vs. non-black men in the present analysis, we found that a first-degree family history of prostate cancer was positively associated with high-grade prostate cancer regardless of race. Past research testing the association between first-degree prostate cancer family history and overall prostate cancer risk in black and white men, though limited to four studies none of which considered tumor aggressiveness, has not found substantial racial differences [9–12], as summarized by a recent review [8]. These four studies [9–12] reported effect sizes in black men that were similar in magnitude to two other studies [17, 18] examining associations between family history and prostate cancer risk in black men only. Hayes reported increased odds of overall prostate cancer in association with positive first-degree family history (OR 3.2, 95% CI 2.0–5.0), but found similar associations for both black men and white men when stratifying by race [11]. The results from studies by Cunningham [black men (OR 1.58, 95% CI 1.05–2.29), non-black men (OR 1.65, 95% CI 1.06–2.15)] [9], Whittemore [black men (OR 3.2, 95% CI 2.0–5.0), white men (OR 1.9, 95% CI 1.9–2.9)] [10] and Sanderson [black men (OR 2.40, 95% CI 1.28–4.51), white men (OR 2.29, 95% CI 1.36–3.85)] [12] also found no definitive differences by race in associations between first-degree family history and overall prostate cancer risk. As such, our study provides some of the first data to suggest that while associations between any positive family history of prostate cancer and high-grade prostate cancer may vary by race, a first-degree family history was associated with high-grade prostate cancer regardless of race. These findings will, however, require validation by other larger studies.

Even in predominantly white populations, relatively few prior studies have examined associations between degree of family history of prostate cancer and risk of aggressive prostate cancer. An analysis of the Swiss screening arm of the European Randomized Study of Screening for Prostate Cancer (ERSPC) by Randazzo did not find a significant association between first-degree prostate cancer family history and high-grade prostate cancer risk, though the results were suggestive (OR 1.52, 95% CI 0.92–1.51). Their lack of statistically significant results could be due to the diagnosis of high-grade prostate cancer in only < 0.5% of the study population and the inclusion of men with a second-degree prostate cancer family history in the reference group, which may have attenuated the associations [19]. Another study by Thomas et al., using data from the multinational REDUCE prostate cancer chemoprevention trial, reported a positive association between a first-degree family history of prostate cancer and high-grade prostate cancer (OR 1.51, 95% CI 1.10–2.06), with a similar association observed for low-grade cancer [4]. These findings may only apply to white men since the study population included an insufficient number of black participants to examine associations in this group separately. Another study in the US, also in a predominantly white population, reported an increased risk of both low-grade (OR 1.75, 95% CI 1.59–1.93) and high-grade (OR 1.66, 95% CI 1.43–1.92) prostate cancer in men with a first-degree family history of prostate cancer [6]. However, another US study reported that family history of prostate cancer was associated with more aggressive tumor characteristics, including seminal vesical invasion (OR 1.91, 95% CI 0.98–3.73) and high tumor stage (OR 1.43, 95% CI 1.00–2.05) [20]. While these studies focused on tumor aggressiveness at diagnosis, a first-degree family history of prostate cancer has also been associated with fatal prostate cancer, with associations reaching statistical significance in two studies (OR 1.72, 95% CI 1.25–2.38 [13] and OR 1.60, 95% CI 1.31–1.97 [21]) and falling short of significance in another study potentially due to low numbers of fatal events (OR 2.97, 95% CI 0.85–10.38 [19]). A Utah study found that having one or more first-degree relatives (OR 2.67, 95% CI 2.45–2.91) or one or more second-degree relatives (OR 1.65, 95% CI 1.50–1.81) deceased from prostate cancer was associated with increased odds of lethal prostate cancer [22]. In summary, although many studies have reported similar associations between family history of prostate cancer and risk of overall and aggressive disease, our findings are some of the first to suggest that associations with family history may be more pronounced for high-grade prostate cancer.

In the present study, a family history of prostate cancer in second-degree relatives only was associated with reduced odds of overall, low-grade and high-grade prostate cancer diagnosis in non-black men. However, caution should be used in interpreting these results as they are based on only six prostate cancer cases diagnosed in non-black men reporting a second-degree family history of the disease. In contrast to these results in non-black men, the direction and magnitude of estimates for overall, low-grade or high-grade prostate cancer did not vary substantially by degree of family history in black men. In line with our findings in black men, a US study conducted in a predominantly white population found an increased risk of prostate cancer in patients with no first-degree family history of prostate cancer and at least one second-degree relative with prostate cancer (OR 1.51, 95% CI 1.47–1.56) [23]. Although larger studies of racially diverse populations are needed to further clarify these relationships, our findings provide some preliminary suggestive evidence that the risk of prostate cancer among men reporting a second-degree family history of the disease may vary by race.

This study relied on self-reported family history, which allows for the potential of recall bias. However, a previous study found an 86% accuracy rate in the self-reporting of family history of prostate cancer in a first-degree relative [24]. Records showed that 14% of the misreported cases of prostate cancer in a relative were in fact due to BPH (benign prostatic hyperplasia) and not malignancy. It is also possible that men reporting a second-degree family history of prostate cancer misreported a non-prostate cancer condition such as BPH rather than prostate cancer in the affected relative [25]. A second study found a similar accuracy rate of 90% for reporting family history of prostate cancer in any degree of relative, also finding that reports of prostate cancer in first-degree relatives were even more accurate than for second- or third-degree relatives [25]. A second limitation involves the 22% of participants with unknown family history due to uncertainty about their family history or missing questionnaire data. Since no association was found between unknown family history of prostate cancer and prostate cancer diagnosis, overall or when stratified by race or grade, it is unlikely that these missing data would have altered our results. Third, it is known that men with a negative prostate biopsy may in fact have prostate cancer detected on a future biopsy, though men in our study had multiple biopsies if clinically indicated, so the risk of this creating a bias is low. Finally, our analysis examined the relationship of family history to prostate cancer aggressiveness among men with an elevated PSA and/or abnormal DRE undergoing prostate biopsy, and therefore, our results may not be generalizable to all men. A strength of this study was the racial diversity of the study population, with black men comprising 59% of the participants, allowing us to test for race interactions. The VA is an equal access healthcare system, which should limit possible racial disparities in prostate cancer screening and detection [26]. Given that prostate cancer screening behavior may be affected by a known family history of the disease, equal access to care may serve to lessen the potential influence of differential screening based on knowledge of family history of prostate cancer.

Given the higher incidence of aggressive prostate cancer in black men compared to other racial groups, there is likely a genetic component to the prostate cancer racial disparity [27] that could be captured by examining family history of the disease. This study sought to address the racial disparity in research regarding the association between prostate cancer family history and prostate cancer diagnosis, overall and by tumor grade, by utilizing a racially diverse, equal access healthcare population comprised of 59% black men. This is one of the few studies to stratify results by tumor aggressiveness, which we chose to study, since it is known that high-grade prostate cancer is more prevalent in the black population. Our results show that men undergoing prostate biopsy with a family history of prostate cancer had increased odds of being diagnosed with a high-grade prostate cancer, but not a low-grade prostate cancer. Upon stratifying by race, we found that the magnitude of this association was stronger and reached statistical significance only in black men, though the trend of positive association was observed in both black and non-black men. However, upon further exploration by degree of family history, we found similar associations between a first-degree family history of prostate cancer and tumor aggressiveness by race, perhaps pointing to a stronger effect of second-degree family history on aggressive prostate cancer in black vs. non-black men, though this should be confirmed by future studies. Though the majority of research in identifying risk variants associated with prostate cancer has focused on men of European ancestry, these variants are being validated and novel variants have been discovered in black men [27–34]. Further research is needed to determine the prevalence of such genetic markers in different racial groups as well as to identify variants preferentially associated with high-grade prostate cancer [35, 36]. Future studies should also assess whether awareness of family history has an impact on these findings by influencing screening and biopsy rates in men with a family history of prostate cancer.

## Supplementary Information

Below is the link to the electronic supplementary material.

Electronic supplementary material 1 (DOCX 13 kb)

Electronic supplementary material 2 (DOCX 13 kb)

## Abbreviations

BMI: Body mass index
BPH: Benign prostatic hyperplasia
DRE: Digital rectal examination
DVAMC: Durham Veterans Affairs Medical Center
FH: Family history
OR: Odds ratio
PC: Prostate cancer
TRUS: Transurethral ultrasound

## References

[1] Siegel RL, Miller KD, Jemal A (2019). Cancer statistics, 2019. CA: Cancer J Clin.

[2] DeSantis CE, Miller KD, Goding Sauer A, Jemal A, Siegel RL (2019). Cancer statistics for African Americans 2019. CA: Cancer J Clin.

[3] Nettey OS, Walker AJ, Keeter MK, Singal A, Nugooru A, Martin IK (2018). Self-reported Black race predicts significant prostate cancer independent of clinical setting and clinical and socioeconomic risk factors. Urol Oncol.

[4] Thomas JA, Gerber L, Moreira DM, Hamilton RJ, Banez LL, Castro-Santamaria R (2012). Prostate cancer risk in men with prostate and breast cancer family history: results from the REDUCE study. J Intern Med.

[5] Hjelmborg JB, Scheike T, Holst K, Skytthe A, Penney KL, Graff RE (2014). The heritability of prostate cancer in the Nordic Twin Study of Cancer. Cancer Epidemiol Biomark Prev.

[6] Chen YC, Page JH, Chen R, Giovannucci E (2008). Family history of prostate and breast cancer and the risk of prostate cancer in the PSA era. Prostate.

[7] Kicinski M, Vangronsveld J, Nawrot TS (2011). An epidemiological reappraisal of the familial aggregation of prostate cancer: a meta-analysis. PLoS ONE.

[8] Mordukhovich I, Reiter PL, Backes DM, Family L, McCullough LE, O'Brien KM (2011). A review of African American-white differences in risk factors for cancer: prostate cancer. Cancer Causes Control: CCC.

[9] Cunningham GR, Ashton CM, Annegers JF, Souchek J, Klima M, Miles B (2003). Familial aggregation of prostate cancer in African-Americans and white Americans. Prostate.

[10] Whittemore AS, Wu AH, Kolonel LN, John EM, Gallagher RP, Howe GR (1995). Family history and prostate cancer risk in black, white, and Asian men in the United States and Canada. Am J Epidemiol.

[11] Hayes RB, Liff JM, Pottern LM, Greenberg RS, Schoenberg JB, Schwartz AG (1995). Prostate cancer risk in U.S. blacks and whites with a family history of cancer. Int J Cancer.

[12] Sanderson M, Coker AL, Logan P, Zheng W, Fadden MK (2004). Lifestyle and prostate cancer among older African-American and Caucasian men in South Carolina. Cancer Causes Control: CCC.

[13] Barber L, Gerke T, Markt SC, Peisch SF, Wilson KM, Ahearn T (2018). Family history of breast or prostate cancer and prostate cancer risk. Clin Cancer Res.

[14] Guerrios-Rivera L, Howard L, Frank J, De Hoedt A, Beverly D, Grant DJ (2017). Is body mass index the best adiposity measure for prostate cancer risk? Results from a veterans affairs biopsy cohort. Urology.

[15] Epstein JI, Zelefsky MJ, Sjoberg DD, Nelson JB, Egevad L, Magi-Galluzzi C (2016). A contemporary prostate cancer grading system: a validated alternative to the gleason score. Eur Urol.

[16] Mucci LA, Hjelmborg JB, Harris JR, Czene K, Havelick DJ, Scheike T (2016). Familial risk and heritability of cancer among twins in Nordic countries. JAMA.

[17] Beebe-Dimmer JL, Drake EA, Dunn RL, Bock CH, Montie JE, Cooney KA (2006). Association between family history of prostate and breast cancer among African-American men with prostate cancer. Urology.

[18] Nemesure B, Wu SY, Hennis A, Leske MC (2013). Family history of prostate cancer in a black population. J Immigr Minor Health.

[19] Randazzo M, Muller A, Carlsson S, Eberli D, Huber A, Grobholz R (2016). A positive family history as a risk factor for prostate cancer in a population-based study with organised prostate-specific antigen screening: results of the Swiss European Randomised Study of Screening for Prostate Cancer (ERSPC, Aarau). BJU Int.

[20] Spangler E, Zeigler-Johnson CM, Malkowicz SB, Wein AJ, Rebbeck TR (2005). Association of prostate cancer family history with histopathological and clinical characteristics of prostate tumors. Int J Cancer.

[21] Rodriguez C, Calle EE, Miracle-McMahill HL, Tatham LM, Wingo PA, Thun MJ (1997). Family history and risk of fatal prostate cancer. Epidemiology.

[22] Albright FS, Stephenson RA, Agarwal N, Cannon-Albright LA (2017). Relative risks for lethal prostate cancer based on complete family history of prostate cancer death. Prostate.

[23] Albright F, Stephenson RA, Agarwal N, Teerlink CC, Lowrance WT, Farnham JM (2015). Prostate cancer risk prediction based on complete prostate cancer family history. Prostate.

[24] King TM, Tong L, Pack RJ, Spencer C, Amos CI (2002). Accuracy of family history of cancer as reported by men with prostate cancer. Urology.

[25] Gaff CL, Aragona C, MacInnis RJ, Cowan R, Payne C, Giles GG (2004). Accuracy and completeness in reporting family history of prostate cancer by unaffected men. Urology.

[26] Graham-Steed T, Uchio E, Wells CK, Aslan M, Ko J, Concato J (2013). 'Race' and prostate cancer mortality in equal-access healthcare systems. Am J Med.

[27] Du Z, Lubmawa A, Gundell S, Wan P, Nalukenge C, Muwanga P (2018). Genetic risk of prostate cancer in Ugandan men. Prostate.

[28] Haiman CA, Chen GK, Blot WJ, Strom SS, Berndt SI, Kittles RA (2011). Characterizing genetic risk at known prostate cancer susceptibility loci in African Americans. PLoS Genet.

[29] Haiman CA, Han Y, Feng Y, Xia L, Hsu C, Sheng X (2013). Genome-wide testing of putative functional exonic variants in relationship with breast and prostate cancer risk in a multiethnic population. PLoS Genet.

[30] Al Olama AA, Kote-Jarai Z, Berndt SI, Conti DV, Schumacher F, Han Y (2014). A meta-analysis of 87,040 individuals identifies 23 new susceptibility loci for prostate cancer. Nat Genet.

[31] Hoffmann TJ, Van Den Eeden SK, Sakoda LC, Jorgenson E, Habel LA, Graff RE (2015). A large multiethnic genome-wide association study of prostate cancer identifies novel risk variants and substantial ethnic differences. Cancer Discov.

[32] Virlogeux V, Graff RE, Hoffmann TJ, Witte JS (2015). Replication and heritability of prostate cancer risk variants: impact of population-specific factors. Cancer Epidemiol Biomark Prev.

[33] Tan SH, Petrovics G, Srivastava S (2018). Prostate cancer genomics: recent advances and the prevailing underrepresentation from racial and ethnic minorities. Int J Mol Sci.

[34] Gusev A, Shi H, Kichaev G, Pomerantz M, Li F, Long HW (2016). Atlas of prostate cancer heritability in European and African-American men pinpoints tissue-specific regulation. Nat Commun.

[35] Helfand BT, Roehl KA, Cooper PR, McGuire BB, Fitzgerald LM, Cancel-Tassin G (2015). Associations of prostate cancer risk variants with disease aggressiveness: results of the NCI-SPORE Genetics Working Group analysis of 18,343 cases. Hum Genet.

[36] Shui IM, Lindstrom S, Kibel AS, Berndt SI, Campa D, Gerke T (2014). Prostate cancer (PCa) risk variants and risk of fatal PCa in the National Cancer Institute Breast and Prostate Cancer Cohort Consortium. Eur Urol.

