# Supplementary material for: Family history of prostate cancer and prostate tumor aggressiveness in black and non-black men;results from an equal access biopsy study
Source: Cancer Causes Control. 2021 Feb 2;32(4):337–46. doi: 10.1007/s10552-020-01389-8 (PMC7946692; doi:10.1007/s10552-020-01389-8)
Supplement: Supplementary file 1 — Electronic supplementary material 1 (DOCX 13 kb) [file 10552_2020_1389_MOESM1_ESM.docx]

**Supplemental Table 1:** Odds ratios and 95% confidence intervals for associations of family history of prostate cancer (overall and by degree) with overall prostate cancer diagnosis at biopsy, among men with no previous prostate biopsy

|  | **Prostate biopsy result** | | | |
| --- | --- | --- | --- | --- |
|  | **Negative** | **Positive** | | |
|  | N_e_/N | N_e_/N | OR (95% CI) | p-value |
| **Family History of PC** |  |  |  |  |
| No | 240/519 | 279/519 | Ref |  |
| Yes | 96/256 | 160/256 |  |  |
| Age-adjusted |  |  | 1.43 (1.06-1.95) | 0.02 |
| Multivariable* |  |  | 1.43 (1.00-2.05) | 0.05 |
| Unknown | 100/223 | 123/223 |  |  |
| Age-adjusted |  |  | 1.07 (0.78-1.47) | 0.67 |
| Multivariable* |  |  | 1.16 (0.80-1.68) | 0.44 |
| **Family History Degree** |  |  |  |  |
| None | 240/519 | 279/519 | Ref |  |
| First | 72/200 | 128/200 |  |  |
| Age-adjusted |  |  | 1.54 (1.10-2.16) | 0.01 |
| Multivariable* |  |  | 1.64 (1.10-2.43) | 0.01 |
| Second | 24/56 | 32/56 |  |  |
| Age-adjusted |  |  | 1.13 (0.65-1.97) | 0.67 |
| Multivariable* |  |  | 0.92 (0.47-1.81) | 0.80 |

*Adjusted for: Age at consent, race, year of consent, PSA (log transformed), DRE, and TRUS volume (log transformed).

Abbreviations: PC=Prostate Cancer; OR=Odds Ratio; CI=Confidence Interval
