# Supplementary material for: Family history of prostate cancer and prostate tumor aggressiveness in black and non-black men;results from an equal access biopsy study
Source: Cancer Causes Control. 2021 Feb 2;32(4):337–46. doi: 10.1007/s10552-020-01389-8 (PMC7946692; doi:10.1007/s10552-020-01389-8)
Supplement: Supplementary file 2 — Electronic supplementary material 2 (DOCX 13 kb) [file 10552_2020_1389_MOESM2_ESM.docx]

**Supplemental Table 2:** Odds ratios and 95% confidence intervals for associations of family history of prostate cancer (overall and by degree) with low-grade and high-grade prostate cancer diagnosis at biopsy, among men with no previous prostate biopsy

|  | **Prostate biopsy result** | | | | | | |
| --- | --- | --- | --- | --- | --- | --- | --- |
|  | **Negative** | **Positive: Low-grade prostate cancer** | | | **Positive: High-grade prostate cancer** | | |
|  | N_e_/N | N_e_/N | OR (95% CI) | p-value | N_e_/N | OR (95% CI) | p-value |
| **Family History of PC** |  |  |  |  |  |  |  |
| No | 240/519 | 202/519 | Ref |  | 77/519 | Ref |  |
| Yes | 96/256 | 104/256 |  |  | 56/256 |  |  |
| Age-adjusted |  |  | 1.29 (0.92-1.80) | 0.14 |  | 1.86 (1.22-2.83) | 0.004 |
| Multivariable* |  |  | 1.33 (0.92-1.94) | 0.13 |  | 1.88 (1.13-3.13) | 0.01 |
| Unknown | 100/223 | 92/223 |  |  | 31/223 |  |  |
| Age-adjusted |  |  | 1.09 (0.77-1.53) | 0.63 |  | 1.01 (0.63-1.64) | 0.96 |
| Multivariable* |  |  | 1.15 (0.78-1.69) | 0.48 |  | 1.18 (0.66-2.09) | 0.58 |
| **Family History Degree** |  |  |  |  |  |  |  |
| None | 240/519 | 202/519 | Ref |  | 77/519 | Ref |  |
| First | 72/200 | 84/200 |  |  | 44/200 |  |  |
| Age-adjusted |  |  | 1.39 (0.96-2.01) | 0.08 |  | 1.98 (1.25-3.15) | 0.004 |
| Multivariable* |  |  | 1.52 (1.01-2.30) | 0.04 |  | 2.19 (1.25-3.82) | 0.006 |
| Second | 24/56 | 20/56 |  |  | 12/56 |  |  |
| Age-adjusted |  |  | 0.98 (0.53-1.84) | 0.96 |  | 1.52 (0.72-3.21) | 0.28 |
| Multivariable* |  |  | 0.85 (0.42-1.73) | 0.66 |  | 1.20 (0.47-3.06) | 0.70 |

*Adjusted for: Age at consent, race, year of consent, PSA (log transformed), DRE, and TRUS volume (log transformed).

Abbreviations: PC=Prostate Cancer; OR=Odds Ratio; CI=Confidence Interval
